# Follikuläres Lymphom der Bindehaut mit aggressiv transformiertem systemischem Befall

**DOI:** 10.1007/s00347-021-01359-8

**Published:** 2021-03-16

**Authors:** V. Schöneberger, F. I. Meyer, P. Lohneis, L. M. Heindl

**Affiliations:** 1 Zentrum für Augenheilkunde, Medizinische Fakultät und Uniklinik Köln, Universität zu Köln, Kerpener Str. 62, 50937 Köln, Deutschland; 2 Klinik für Radiologie, Medizinische Fakultät und Uniklinik Köln, Universität zu Köln, Köln, Deutschland; 3 Pathologisches Institut, Medizinische Fakultät und Uniklinik Köln, Universität zu Köln, Köln, Deutschland; 4 Centrum für Integrierte Onkologie (CIO) Aachen-Bonn-Köln-Düsseldorf, Köln, Deutschland

## Konjunktivale Lymphome

Lymphome der okulären Adnexe sind eine seltene Manifestation der Non-Hodgkin-Lymphome (NHL). Diese machen zwar nur 1–2 % aller NHL aus, stellen jedoch die häufigste Malignommanifestation der Orbita dar. Die okulären Adnexe umfassen das Augenlid, die Tränendrüse, das orbitale Gewebe und die Konjunktiva. Letztere ist in etwa 25 % der Fälle betroffen [1]. Konjunktivale Lymphome präsentieren sich typischerweise zwischen dem 50. und 70. Lebensjahr als eine schmerzlose lachsfarbene Schwellung der Konjunktiva [2]. Eine chirurgische Biopsie zur eindeutigen Diagnose ist unumgänglich. Der histopathologische Subtyp und das Stadium des konjunktivalen Lymphoms stellen die wichtigsten Prädiktoren für das Outcome dar [3, 4]. Am häufigsten finden sich bei konjunktivalen Lymphomen die Low-Grade-Formen (Tab. 1).

**Table 1:** 

Histologie	Grading	Anteil der konjunktivalen Lymphome (%)
Extranodales Marginalzonenlymphom	Low-Grade-Tumor	80
Follikuläres Lymphom	Low-Grade-Tumor	8
Diffus großzelliges B‑Zell-Lymphom	High-Grade-Tumor	3
Mantelzelllymphom	High-Grade-Tumor	3
T‑Zell-Non-Hodgkin-Lymphom	High-Grade-Tumor	2

Das niedrigmaligne NHL der Bindehaut liegt meist isoliert vor (76–90 %) und kann meist durch eine Bestrahlung geheilt werden [2]. Nach Diagnosestellung erfolgt die Vorstellung bei einem Onkologen zum weiteren Staging und Ausschluss einer systemischen Beteiligung, welche bei High-Grade-Lymphomen häufiger zu beobachten ist [1]. Aufgrund der erforderlichen interdisziplinären Zusammenarbeit zwischen Augenklinik, Radiologie, Hämatoonkologie und Pathologie sollte eine gemeinsame Fallbesprechung in der örtlichen Tumorkonferenz stattfinden [3].

Wir möchten im Folgenden einen seltenen Fall eines follikulären Lymphoms der Konjunktiva mit aggressiver molekularer Transformation und systemischer Beteiligung präsentieren.

## Kasuistik

**Figure 1:**
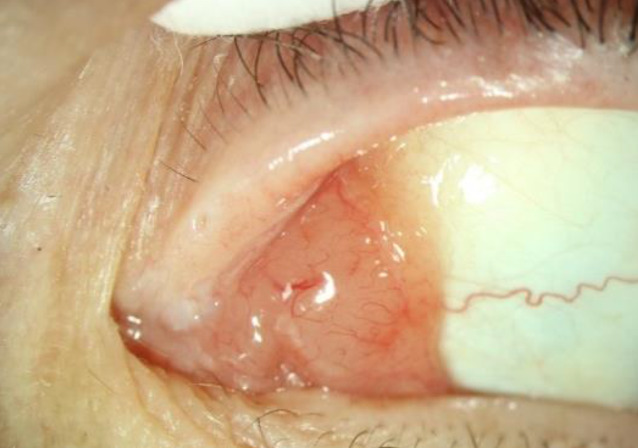

Eine 55-jährige Patientin stellte sich vor drei Jahren erstmalig mit einer schmerzlosen, juckenden Schwellung am linken Auge vor, die seit einigen Monaten zunehmend war. Eine B‑Symptomatik wurde verneint. In der Untersuchung mittels Spaltlampe zeigte sich am linken Auge eine lachsfarbene, leicht erhabene Läsion an der medialen, karunkelnahen Bindehaut (Abb. 1 und 2). Der Vorderabschnitt war reizfrei, die bestkorrigierte Sehschärfe betrug 1,0 beidseits.

**Figure 2:**
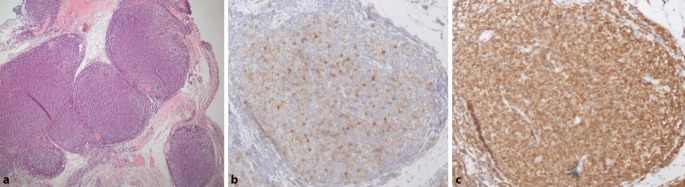

## Weiteres Prozedere

Zur weiterführenden Diagnostik wurde zeitnah eine chirurgische Biopsie geplant. Die histopathologische Diagnostik nach der Plica-Exzision erbrachte ein B‑NHL vom Typ eines follikulären Lymphoms Grad 1–2 der Konjunktiva mit einem Ki67-Proliferationsindex von 10–15 %.

**Figure 3:**
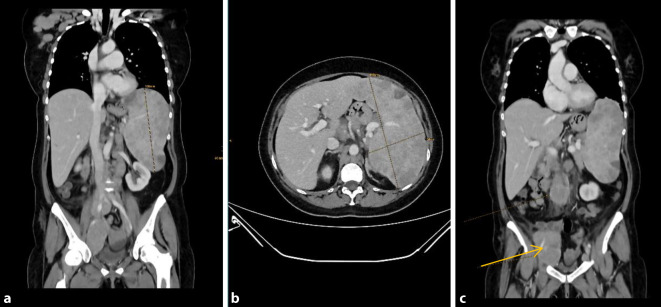

Zur Abgrenzung eines primär konjunktivalen Lymphoms oder einer konjunktivalen Manifestation eines nodalen Lymphoms erfolgte eine klinisch-hämatologische Untersuchung in der onkologischen Ambulanz. Das radiologische Staging mittels Computertomographie (CT) ergab ein *follikuläres Lymphom Grad 1–2 im Stadium IV A E*, aufgrund eines disseminierten Lymphknotenbefalls und einer massiven Hepatosplenomegalie mit einer Milzgröße von 10 × 16 × 19 cm (normalerweise 4 × 7 × 11 cm; Abb. 3a, b), fehlender B‑Symptomatik (A) und extranodalem Befall (E) der Konjunktiva. Die Histologie der konjunktivalen Biopsie war ausreichend, um eine systemische Therapie mittels R‑CHOP (Rituximab, Cyclophosphamid, Doxorubicin, Vincristin und Prednisolon) einzuleiten.

**Figure 4:**
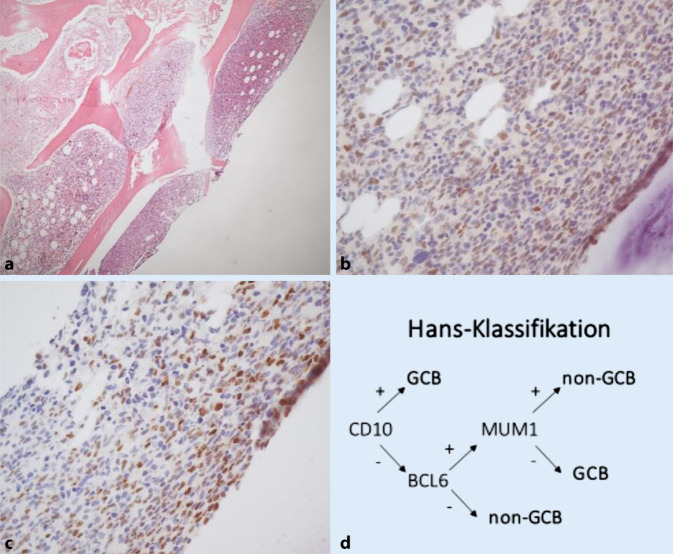

Jedoch wurde die Patientin nur wenige Tage später mit ausgeprägter B‑Symptomatik (Fieber, Schüttelfrost, Tachykardien) stationär in einer externen Uniklinik aufgenommen. Dort erfolgte eine CT-gesteuerte Punktion eines iliakalen Lymphknotens (Abb. 3c) und des Knochenmarks. Daraus ergab sich die Diagnose eines aggressiven NHL vom Typ des *diffus großzelligen B‑Zell-Lymphoms bei Ann-Arbor IV B* (B-Symptomatik). Die immunhistochemischen Färbungen zeigten nun im Gegensatz zur Probebiopsie der Konjunktiva eine Negativität für bcl2 und CD10. Bei Expression von BCL6 und MUM1 handelte es sich nach der Hans-Klassifikation [5] um einen Non-GCB(Germinal-Center-B-Cell-like)-Typ (Abb. 4b–d). Es erfolgte eine Vorphasentherapie mit Cyclophosphamid und Dexamethason mit anschließender Therapie nach dem R‑CHOP-Schema. In der Verlaufs-CT konnte daraufhin eine deutliche Regredienz der bekannten Lymphommanifestationen, einschließlich des okulären Befundes, festgestellt werden.

## Diskussion: sekundär simultanes DLBCL oder primär transformiertes follikuläres Lymphom?

Sowohl beim diffus großzelligen B‑Zell-Lymphom (DLBCL) als auch beim transformierten follikulären Lymphom (t-FL) handelt es sich um aggressive Lymphome, ganz im Gegensatz zum indolenten follikulären Lymphom. Im Allgemeinen ist das follikuläre Lymphom das zweithäufigste diagnostizierte Lymphom in den Vereinigten Staaten und Westeuropa und transformiert in etwa 22 % innerhalb von 5 Jahren. Zu den follikulären Lymphomen der okulären Adnexen liegen nur wenige Daten vor. Rasmussen et al. beschrieben in einer internationalen multizentrischen retrospektiven Studie über einen Zeitraum von 30 Jahren lediglich eine Transformationsrate von 3 % bei follikulären Lymphomen der okulären Adnexe. Eine Transformation geht einher mit einer schnelleren Progression der Lymphadenopathie, B‑Symptomen, erhöhtem LDH-Wert, Hyperkalzämie und einem Nichtansprechen auf die Initialtherapie [6–9].

Follikuläre Lymphome leiten sich von B‑Zellen des Keimzentrums („germinal center B‑cells“, GCB) ab. Daher ist es nicht verwunderlich, dass die allermeisten t‑FL einen GCB-Phänotyp haben (89 %) [5]. Allgemein kann ein t‑FL morphologisch nicht von einem De-novo-DLBCL unterschieden werden [10]. In unserem Fall wurde daher zunächst bei vorliegendem Non-GCB-Typ und der schnellen Progression von einer zweiten, unabhängigen Lymphommanifestation in kürzestem Zeitraum ausgegangen. Im Gegensatz zu einem transformierten Lymphom ist das Ansprechen auf eine Chemotherapie bei einer De-novo-Erkrankung besser und die Überlebenszeit länger [11].

Um die Frage abschließend zu klären, ob es sich um ein *sekundär simultanes DLBCL oder primär transformiertes follikuläres Lymphom *handelt, wurde eine IgH-Klonalitätsanalyse durchgeführt. Die Analyse beruht methodisch auf einer PCR-Fragmentanalyse und zeigt spezifische Rearrangements in der leichten und schweren Kette des Immunglobulin-G-Gens. Dadurch kann die Abstammung von einer gemeinsamen Ursprungszelle (Klonalität) molekularpathologisch festgestellt werden. Die Untersuchung zeigte überraschenderweise übereinstimmende DNA-Fragmentlängen in der Probebiopsie der Karunkula und des iliakalen Lymphknotens, wodurch schlussendlich die Diagnose eines tFL vom selteneren ABC-Typ getroffen werden konnte.

Dieser Fall zeigt auf, dass die Zuordnung zur Ursprungszelle („cell of origin“) wichtig ist, um eine klare Diagnose zu treffen und entsprechend auch die Prognose und das Ansprechen auf eine zielgerichtete Therapie vorhersagen zu können. Aktuell sieht man keine schlechtere Prognose beim ABC-Typ im Vergleich zum GCB-Typ, jedoch liegen aufgrund der Seltenheit des Befundes nur wenige Daten vor [10]. Die Feststellung, dass eine Untergruppe der t‑FL dem ABC-Subtyp angehört, ist besonders faszinierend, da sie die Frage aufwirft, ob diese Patienten überhaupt Kandidaten für eine gezielte Beeinflussung des B‑Zell-Rezeptor-Signalwegs wären. So könnten zielgerichtete Wirkstoffe wie Ibrutinib und Lenalidomid bei ABC-tFL untersucht werden. Aktuell läuft eine Phase II-Studie bei refraktären oder rezidivierten DCBLC mit Non-GCB-Typ (registriert unter #NCT02077166) [12].

## Weiterer Verlauf

**Figure 5:**
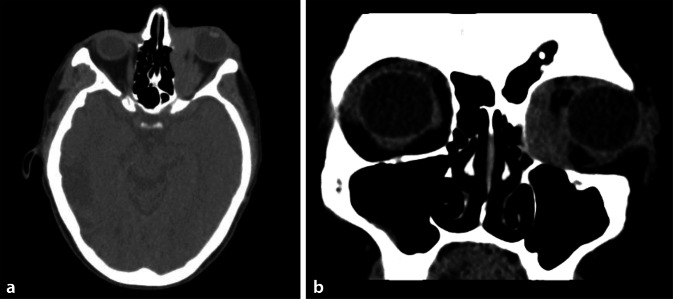

Ende des letzten Jahres stellte sich uns die Patientin erneut bei dringlichem Verdacht auf ein Lymphomrezidiv intraorbital links vor. In der CT waren eine orbitale Raumforderung links (Abb. 5) und geschwollene Lymphknoten mediastinal und rechts axillär zu sehen. Eine axilläre Lymphknotenbiopsie erbrachte jedoch einen negativen Befund.

Durch die ausgeprägte orbitale Raumforderung kam es bei der Patientin zu einem Exophthalmus und einer Exophorie mit zunehmenden Doppelbildern. Der Visus verschlechterte sich linksseitig bestkorrigiert auf 0,4. Die inzisionale Biopsie mittels eines transkonjunktivalen anterioren Zugangs erbrachte nach histologischer und molekularbiologischer Untersuchung erneut den Befund eines *niedrigmalignen, follikulären NHL der B‑Zellreihe, *das als Rezidiv des bekannten follikulären Lymphoms drei Jahre zuvor zu werten war. Es erfolgte umgehend die Einleitung einer Rezidivtherapie mit Rituximab und Bendamustin. Ein halbes Jahr später erfolgte ein CT-Staging, das eine erhebliche Größenregredienz aller vorbekannten Lymphommanifestationen zeigte. Die Patientin befindet sich weiterhin in der Tumornachsorge, aktuell gehe es ihr gut.

## Fazit für die Praxis

- Die Transformation eines Lymphoms ist selten, unter Beteiligung des Auges sogar ungewöhnlich.
- Hier wird ein solcher Fall mit aggressiver histologischer Transformation von einem ursprünglichen (indolenten) Low-Grade-Lymphom beschrieben.
- Eine Transformation eines indolenten Lymphoms in ein aggressives Lymphom geht mit einer schlechteren Prognose einher.

## References

[1] Kirkegaard MM (2015). Malignant lymphoma of the conjunctiva. Surv Ophthalmol.

[2] Tanenbaum RE (2019). Classification, diagnosis, and management of conjunctival lymphoma. Eye Vis (Lond).

[3] Kakkassery V (2020). Ocular lymphoma: precise diagnostics and classification as key for successful personalized treatment. Ophthalmologe.

[4] Kakkassery V (2015). Lymphoma of the ocular adnexa. Ophthalmologe.

[5] Hans CP (2004). Confirmation of the molecular classification of diffuse large B-cell lymphoma by immunohistochemistry using a tissue microarray. Blood.

[6] Freedman AS (2005). Biology and management of histologic transformation of indolent lymphoma. Hematology Am Soc Hematol Educ Program.

[7] Swerdlow SH (2016). The 2016 revision of the World Health Organization classification of lymphoid neoplasms. Blood.

[8] Bastion Y (1997). Incidence, predictive factors, and outcome of lymphoma transformation in follicular lymphoma patients. J Clin Oncol.

[9] Rasmussen PK (2014). Ocular adnexal follicular lymphoma: a multicenter international study. JAMA Ophthalmol.

[10] Kridel R (2015). Cell of origin of transformed follicular lymphoma. Blood.

[11] Davies AJ (2007). Radioimmunotherapy for B-cell lymphoma: Y90 ibritumomab tiuxetan and I(131) tositumomab. Oncogene.

[12] Goy A (2019). Ibrutinib plus lenalidomide and rituximab has promising activity in relapsed/refractory non-germinal center B-cell-like DLBCL. Blood.

